# Post-acute care use patterns among Hospital Service Areas by older adults in the United States: a cross-sectional study

**DOI:** 10.1186/s12913-021-06159-z

**Published:** 2021-02-25

**Authors:** Julianna M. Dean, Kimberly Hreha, Ickpyo Hong, Chih-Ying Li, Daniel Jupiter, John Prochaska, Timothy Reistetter

**Affiliations:** 1 University of Houston-Clear Lake, 2700 Bay Area Blvd, Houston, TX 77058 USA; 2 University of Texas Medical Branch, 301 University Blvd, Galveston, TX 77555 USA; 3 Yonsei University, 135 Backun Hall, Yonsei Univroad1, Wonju, Gangwon-do 26493 South Korea; 4 University of Texas Health San Antonio, 7703 Floyd Curl Dr, San Antonio, TX 78229 USA

**Keywords:** Post-acute care, Hospital Service Areas, Stroke, Access to care, Geographic variation

## Abstract

**Background:**

Despite the success of stroke rehabilitation services, differences in service utilization exist. Some patients with stroke may travel across regions to receive necessary care prescribed by their physician. It is unknown how availability and combinations of post-acute care facilities in local healthcare markets influence use patterns. We present the distribution of skilled nursing, inpatient rehabilitation, and long-term care hospital services across Hospital Service Areas among a national stroke cohort, and we describe drivers of post-acute care service use.

**Methods:**

We extracted data from 2013 to 2014 of a national stroke cohort using Medicare beneficiaries (174,498 total records across 3232 Hospital Service Areas). Patients’ ZIP code of residence was linked to the facility ZIP code where care was received. If the patient did not live in the Hospital Service Area where they received care, they were considered a “traveler”. We performed multivariable logistic regression to regress traveling status on the care combinations available where the patient lived.

**Results:**

Although 73.4% of all Hospital Service Areas were skilled nursing-only, only 23.5% of all patients received care in skilled nursing-only Hospital Service Areas; 40.8% of all patients received care in Hospital Service Areas with only inpatient rehabilitation and skilled nursing, which represented only 18.2% of all Hospital Service Areas. Thirty-five percent of patients traveled to a different Hospital Service Area from where they lived. Regarding “travelers,” for those living in a skilled nursing-only Hospital Service Area, 49.9% traveled for care to Hospital Service Areas with only inpatient rehabilitation and skilled nursing. Patients living in skilled nursing-only Hospital Service Areas had more than five times higher odds of traveling compared to those living in Hospital Service Areas with all three facilities.

**Conclusions:**

Geographically, the vast majority of Hospital Service Areas in the United States that provided rehabilitation services for stroke survivors were skilled nursing-only. However, only about one-third lived in skilled nursing-only Hospital Service Areas; over 35% traveled to receive care. Geographic variation exists in post-acute care; this study provides a foundation to better quantify its drivers. This study presents previously undescribed drivers of variation in post-acute care service utilization among Medicare beneficiaries—the “traveler effect”.

**Supplementary Information:**

The online version contains supplementary material available at 10.1186/s12913-021-06159-z.

## Background

Every 40 s, someone in the United States (US) suffers a stroke; over 795,000 strokes occur each year in the US [1]. Among those 65 and older stroke was the fourth leading cause of death in 2016 [2], and it is the chronic condition with the highest total spending per capita for fee-for-service Medicare beneficiaries [2]. A stroke can cause acute impairments that affect the way a person functions in daily life [3]. Even after acute intervention (like tissue plasminogen activator—tPA), residual deficits often require post-acute care (PAC) services to address these impaired functional skills, increase independence, and ultimately help patients return to home or community [3].

Despite the success of rehabilitation services for stroke, differences in service utilization exist across the US [4–6]. The June 2018 Medicare Payment Advisory Commission (MedPAC) Report to Congress recognizes four PAC provider types: inpatient rehabilitation facilities (IRFs), skilled nursing facilities (SNFs), home health agencies (HHAs) and long-term care hospitals (LTCHs) [7]. Geographic variation in the use of these PAC services exists across the US, where patients living in some regions utilize services more often than those living in other regions [4]. For example, patients with stroke use PAC services ranged from 62.6% in the East and West South Central states to 74.5% in the New England area [4]. Although it is unclear what drives the differences in regional use, potential factors may include availability of PAC facilities, patient characteristics and clinical diagnoses and severity, provider availability, and current reimbursement programs [4, 8].

Another potential reason for regional use differences is that some patients with stroke may travel across regions to receive necessary care prescribed by their physician. Hospital Service Areas (HSAs) are geographic healthcare regions designed to quantify boundaries of an area where Medicare beneficiaries received acute care services [9]. These regions can define the number and combinations of PAC facilities within each geographic area. Although other administrative boundaries exist, we chose HSAs in this study as HSAs reflect healthcare markets larger than primary care service areas and smaller than Hospital Referral Regions.

Currently, the Bundled Payments for Care Improvement (BPCI) Initiative provides payment based on a pre-defined group of health services (e.g., surgery, acute stays, post-acute rehabilitation) per episode of care [10]. However, the BPCI does not consider the PAC providers’ specific geographic location. Thus, the BPCI may not account for patients who travel to use PAC services outside of their residential area [11]. While the BPCI encourages providers to reduce overused services, the model may incentivize the providers to underuse services or reduce levels of care, as the providers will keep the unused bundle dollars [12]. A 5–15% reduction in PAC services use was reported after the introduction of BPCI [10]. Patients may choose to travel so they can receive lower-cost care provision. Such financial incentives may influence the level of care provided and the numbers of traveling patients [8, 13].

Additionally, Value-Based Purchasing (VBP) programs incentivize quality of care over quantity of services, and these programs exist across most Medicare settings [14]. However, regardless of the quality of care provided, some beneficiaries may have risk factors that affect their health. In 2016, the Office of the Assistant Secretary for Planning and Evaluation (ASPE) of the US Department of Health and Human Services published a Report to Congress that highlighted social risk factors and their effect on VBP programs [14]. These social risk factors include socioeconomic position, factors in the cultural context such as race and nativity, gender, social relationships, and residential and community factors [14]. Although it may not be clear exactly why beneficiaries travel outside their geographic region to receive care, traveling patterns may affect reported health outcomes in certain areas to which beneficiaries with high social risk factors travel to receive care. In fact, provider location has been cited as a non-clinical factor that influences PAC use patterns [15], and distance to provider has been used as an instrumental variable to capture this construct in analyses across health outcomes in IRFs versus SNFs [16].

It is unknown how the availability and combination of PAC facility types in HSAs influence patterns of PAC use within and among HSAs. Also, little is known about the demographic characteristics of patients receiving care in a different HSA from where they live. Thus, the purpose of this study is to: 1) identify the number and combination of PAC facilities in each HSA in the US that provided stroke rehabilitation; 2) determine the percentage of stroke survivors who received PAC in the same HSA as that in which they reside, and detail the facility combinations present in those HSAs; 3) determine the percentage of stroke survivors who received care in a different HSA from that in which they reside (i.e., “travelers”), and detail the combination of facility patterns of where they reside and where they received care; 4) identify the characteristics of travelers and factors that are associated with traveling to receive PAC services.

As stroke is a common condition seen across all PAC facilities [17] we used this cohort to understand what drives individuals to travel or to receive care where they reside. We hypothesized that patients travel to different HSAs from which they live to receive necessary PAC services, and that more severe patients requiring higher levels of care may be more likely to travel. For example, a patient may travel to a neighboring HSA to receive more intensive IRF care if IRF services meet their needs but cannot be provided in their resident HSA. This study will provide a foundation to better quantify drivers of geographic variation in PAC use across the nation.

## Methods

### Aim, design, and study sample

The aim of this study is to determine how the availability and combinations of PAC facilities in local healthcare markets influence rehabilitation use patterns. We accomplish this by presenting the distribution of SNF, IRF, and LTCH services across HSAs and by identifying variables associated with the odds of traveling outside of the HSA in which a patient lives for PAC services.

This study is a retrospective cross-sectional analysis of 100% Medicare records. Inclusion criteria were an acute stay from January 1, 2013 to December 31, 2014, with a stroke identified by a diagnosis-related group (DRG) code 061–066 (*N* = 675,417). We excluded patients who did not have continuous Medicare coverage for 12 months before and 6 months after the acute admission, a commonly used step to track patients over time [18, 19] (removed *n* = 236,718 (35.0%); *N* = 438,699). Only the first PAC service after the first acute stay was analyzed for each patient [20] (removed *n* = 32,632 (7.4%); *N* = 406,067), and service providers were restricted to IRFs, LTCHs, and SNFs (removed 229,683 (56.6%); *N* = 176,384). Home health services were excluded from analysis, as providers can routinely cover broader distances than HSAs, the highest rates of use are in rural areas [17], and literature suggests significant variation and instability among the market and services provided [21]. Patients were also excluded who had missing facility HSA numbers (removed *n* = 513 (0.3%); *N* = 175,871), whose residence ZIP code did not map to an established HSA (removed *n* = 109 (0.1%); *N* = 175,762), or whose HSA of residence contained no treated patients (removed 1264 (0.7%); final *N* = 174,498). The authors had a data use agreement with Medicare, and this study was approved by their university’s Institutional Review Board.

### Variables

We used HSAs to quantify the heterogeneity and patterns of PAC use [9]. Numbers of HSAs where patients resided and where they received care were identified by linking patient and facility ZIP codes from Medicare files to a crosswalk file containing HSA numbers. Patients admitted to PAC in 2013 were linked with the 2013 crosswalk file and those admitted in 2014 were linked with the 2014 crosswalk [22].

Combinations of facility types for each HSA were identified by determining if any patients in the HSA were treated in the specific facility types. For example, in an HSA that treated 10 patients in IRFs, 0 patients in LTCHs, and 50 patients in SNFs, we assumed the HSA lacked an LTCH. Facility combinations were calculated and reported for each patient in both the HSA where they resided and that where they received care.

A variable was created to show if the patient lived in the same HSA in which they received care. Patients were identified as travelers if they received care in an HSA different from the HSA in which they lived. For travelers, the change in facility combination was reported. For example, if a patient lived in an IRF-only HSA and received care in an IRF-LTCH-SNF combination HSA, the change combination would be IRF-only to IRF-LTCH-SNF.

Covariates used in multivariable logistic regression were categorical and consisted of demographic and clinical factors: age in years (21–64, 65–69, 70–74, 75–79, 80–84, 85+), race (non-Hispanic white, Hispanic, non-Hispanic black, other), sex (male, female), stroke type (ischemic, hemorrhagic), acute length of stay in days (0–3, 4–7, 8–11, 12–25, 26+), dual eligibility (yes/no), and intensive care unit (ICU)/coronary care unit (CCU) stay (yes/no).

### Statistical analysis

Counts and frequencies were calculated for the number of each of the three facility types (IRFs, SNFs, and LTCHs) in each HSA and the combination of facility types for each HSA. This was done both for HSAs in which each patient lived and in which each patient received care. For travelers, counts and frequencies of facility combinations changes were described. We used multivariable logistic regression to regress traveling status (yes/no) on the care combinations available where the patient lived, controlling for the covariates described above. The findings were presented with odds ratios (OR) with 95% confidence intervals (CIs). All data management and statistical procedures were conducted with R version 3.5.3. Maps were created using ArcMap 10.7.

## Results

### Descriptive characteristics

**Fig. 1:**
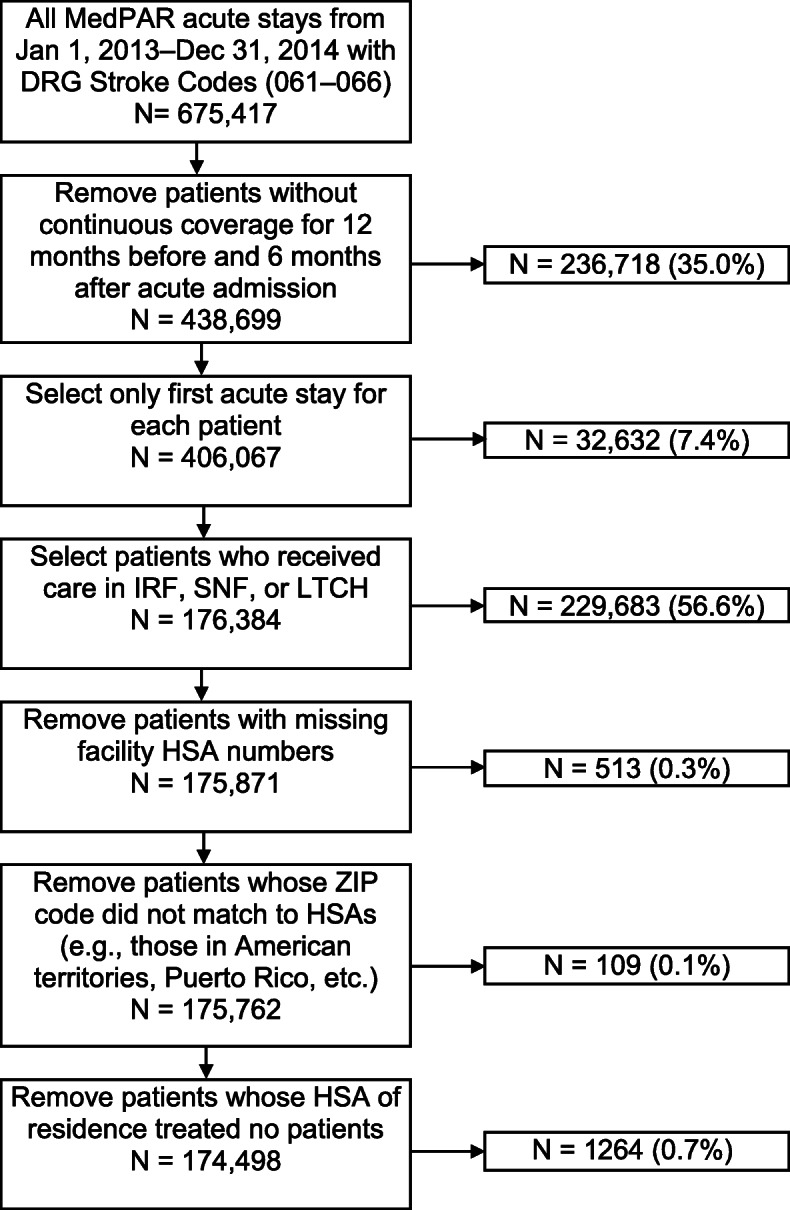
Flowchart of cohort selection. *DRG* diagnosis-related group, *HSA* Hospital Service Area, *IRF* inpatient rehabilitation facility, *LTCH* long-term care hospital, *SNF* skilled nursing facility

**Fig. 2:**
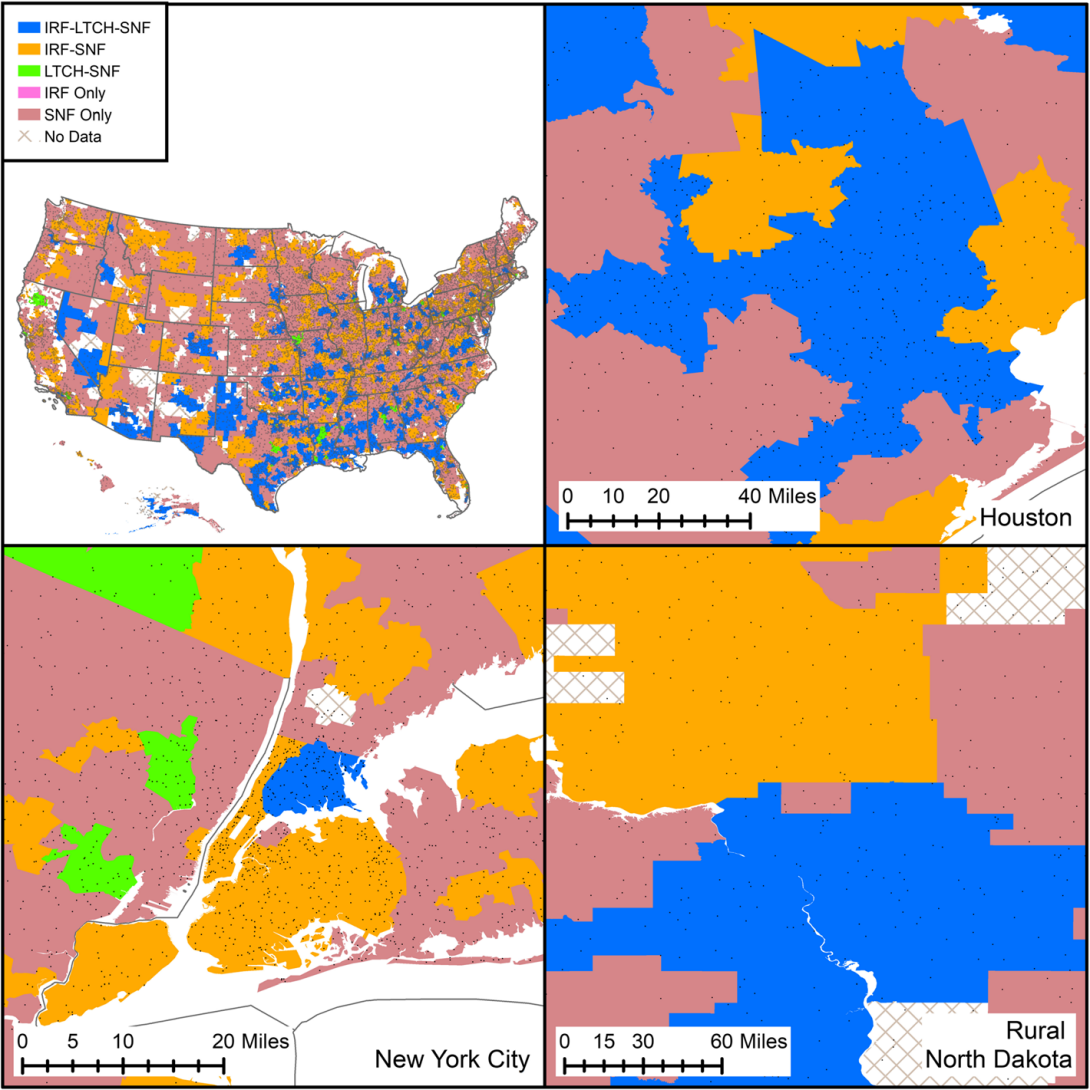
Map of US Hospital Service Areas and of select areas by facility combination type, 2014. For the US, one dot = 5000 people; for all other cities, one dot = 1000 people. *IRF* inpatient rehabilitation facility, *LTCH* long-term care hospital, *SNF* skilled nursing facility. Created by authors using ArcMap 10.7

There were 174,498 patients who met the inclusion/exclusion criteria; 76,469 (43.8%) received care in IRFs, 95,740 (54.9%) in SNFs, and 2289 (1.3%) in LTCHs. The sample contained 3232 unique HSAs (SNF-only: 2377 [73.4%]; IRF-SNF: 588 [18.2%]; IRF-LTCH-SNF: 212 [6.6%]; LTCH-SNF: 56 [1.7%]; IRF-only: 3 [0.1%]). We removed 513 patients who were missing facility HSA numbers, 109 patients whose ZIP code of residence did not match to an HSA (e.g., those in American territories), and 1264 patients whose HSA of residence did not treat patients. Figure 1 presents a flowchart of the cohort selection. If an HSA treated no patients, we were unable to determine the combination of facilities available using our method. However, it is important to note that not all HSAs may have facilities established or available for stroke rehabilitation. Patients received care in HSAs with five different combinations of facilities: SNF-only (40,979; 23.5%), LTCH-SNF (3621; 2.1%), IRF-only (128; 0.1%), IRF-SNF (71,257; 40.8%), and IRF-LTCH-SNF (58,513; 33.5%). Figure 2 maps these facility combinations for each HSA in the US and select cities.

Table 1 presents the demographic characteristics and top 10 most prevalent hierarchical condition categories (HCCs) stratified by traveling status and by PAC service type. Characteristics of additional HCCs can be found in the supplemental material [see Additional file 1]. Ischemic stroke (88.8%) was more common than hemorrhagic (11.2%). Compared to other facility types, SNFs treated a little over half of ischemic (84,008; 54.6%) and hemorrhagic (11,732; 56.6%) patients; IRFs treated 44.1% of ischemic (67,886) and 41.4% of hemorrhagic (8583) patients. The majority of patients were female (104,797; 60.1%), white (137,592; 78.9%), not dual eligible (113,051; 76.2%), and had an ICU/CCU stay (97,069; 55.63%).

**Table 1:** Patient demographics and clinical characteristics by post-acute care service type and traveling status

	TOTAL COHORT			NON-TRAVELERS			TRAVELERS		
	***N*** = 174,498			***N*** = 113,284			***N*** = 61,214		
Variables	IRF (***n*** = 76,469)	LTCH (***n*** = 2289)	SNF (***n*** = 95,740)	IRF (***n*** = 43,089)	LTCH (***n*** = 986)	SNF (***n*** = 69,209)	IRF (***n*** = 33,380)	LTCH (***n*** = 1303)	SNF (***n*** = 26,531)
**Age, yrs**									
**21–64**	6378 (8.34)	257 (11.23)	4571 (4.77)	3647 (8.46)	116 (11.76)	3283 (4.74)	2731 (8.18)	141 (10.82)	1288 (4.85)
**65–69**	9879 (12.92)	276 (12.06)	5987 (6.25)	5378 (12.48)	103 (10.45)	4190 (6.05)	4501 (13.48)	173 (13.28)	1797 (6.77)
**70–74**	12,662 (16.56)	346 (15.12)	9107 (9.51)	6817 (15.82)	121 (12.270	6478 (9.36)	5845 (17.510	225 (17.27)	2629 (9.91)
**75–79**	14,052 (18.38)	365 (15.95)	13,443 (14.04)	7684 (17.83)	166 (16.84)	9516 (13.75)	6368 (19.08)	199 (15.27)	3927 (14.80)
**80–84**	14,578 (19.06)	404 (17.65)	19,269 (45.29)	8330 (19.33)	199 (20.18)	13,937 (20.14)	6248 (18.72)	205 (15.73)	5332 (20.09)
**85+**	18,920 (24.74)	641 (28.00)	43,363 (45.29)	11,233 (26.06)	281 (28.50)	31,805 (45.96)	7687 (23.03)	360 (27.63)	11,558 (43.56)
**Sex**									
**Male**	34,464 (45.01)	1037 (45.30)	34,200 (35.72)	19,006 (44.11)	445 (45.13)	24,670 (35.65)	15,458 (46.31)	592 (45.43)	9530 (35.92)
**Female**	42,005 (54.93)	1252 (54.70)	61,540 (64.28)	24,083 (55.89)	541 (54.87)	44,539 (64.35)	17,922 (53.69)	711 (54.56)	17,001 (64.08)
**Race**									
**Non-Hispanic White**	59,198 (77.41)	1531 (66.89)	76,863 (80.28)	32,452 (75.31)	601 (60.95)	55,762 (80.06)	26,746 (80.13)	930 (71.14)	21,101 (79.53)
**Non-Hispanic Black**	10,517 (13.75)	460 (20.10)	11,817 (12.34)	6755 (15.68)	255 (25.86)	8523 (12.31)	3762 (11.27)	205 (15.98)	3294 (12.42)
**Hispanic**	4029 (5.27)	207 (9.04)	4097 (4.28)	2422 (5.62)	94 (9.53)	2943 (4.25)	1607 (4.81)	113 (8.67)	1154 (4.35)
**Other**	2725 (3.56)	91 (3.98)	2963 (3.09)	1460 (3.39)	36 (3.65)	1981 (2.86)	1265 (3.79)	55 (4.21)	982 (3.70)
**Acute LOS, days**									
**0–3**	31,773 (41.55)	304 (13.28)	28,105 (29.36)	18,755 (43.53)	136 (13.79)	20,941 (30.26)	13,018 (38.99)	168 (12.89)	7164 (27.00)
**4–7**	34,975 (45.74)	814 (35.56)	46,218 (48.27)	19,233 (44.64)	357 (36.21)	33,376 (48.22)	15,742 (47.16)	457 (35.07)	12,842 (48.40)
**8–11**	7007 (9.16)	562 (24.55)	13,139 (13.72)	3727 (8.65)	256 (25.96)	9290 (13.42)	3280 (9.83)	306 (23.48)	3849 (14.51)
**12–25**	2607 (3.41)	549 (23.98)	7662 (8.00)	1324 (3.07)	217 (22.00)	5199 (7.51)	1283 (3.84)	332 (25.48)	2463 (9.23)
**26+**	107 (0.14)	60 (2.62)	616 (0.64)	50 (0.12)	20 (2.03)	403 (0.58)	57 (0.17)	40 (3.07)	213 (0.80)
**Stroke Type**									
**Ischemic**	67,886 (88.76)	1888 (82.48)	84,008 (87.75)	38,617 (89.62)	822 (83.37)	60,897 (87.99)	29,269 (87.68)	1066 (81.81)	23,111 (87.11)
**Hemorrhagic**	8583 (11.22)	401 (17.52)	11,732 (12.25)	4472 (10.38)	164 (16.63)	8312 (12.01)	4111 (12.32)	237 (18.19)	3420 (12.89)
**Dual Eligibility**									
**No**	61,999 (81.08)	1528 (66.75)	69,524 (72.62)	34,654 (80.42)	647 (65.62)	49,938 (72.16)	27,345 (81.92)	881 (67.61)	19,586 (73.82)
**Yes**	14,470 (18.92)	761 (33.25)	26,216 (27.38)	8435 (19.58)	339 (34.38)	19,271 (27.84)	6035 (18.08)	422 (32.39)	6945 (26.17)
**ICU/CCU Stay**									
**No**	30,764 (40.23)	669 (29.23)	45,996 (48.04)	18,067 (41.93)	287 (29.11)	33,812 (48.85)	12,697 (38.04)	382 (29.32)	12,184 (45.92)
**Yes**	45,705 (59.77)	1620 (70.77)	49,744 (51.96)	25,022 (58.07)	699 (70.89)	35,397 (51.15)	20,683 (61.96)	921 (70.68)	14,347 (54.08)
**Hemiplegia/Hemiparesis**									
**No**	40,558 (0.23)	1040 (0.01)	59,791 (0.34)	23,453 (0.21)	448 (0.00)	43,641 (0.39)	17,105 (0.28)	592 (0.01)	16,150 (0.26)
**Yes**	35,911 (0.21)	1249 (0.01)	35,949 (0.21)	19,636 (0.17)	538 (0.00)	25,568 (0.23)	16,275 (0.27)	711 (0.01)	10,381 (0.17)
**Specified Heart Arrhythmias**									
**No**	51,415 (0.29)	1241 (0.01)	57,047 (0.33)	29,079 (0.26)	559 (0.00)	41,335 (0.36)	22,336 (0.36)	682 (0.01)	15,712 (0.26)
**Yes**	25,054 (0.14)	1048 (0.01)	38,693 (0.22)	14,010 (0.12)	427 (0.00)	27,874 (0.25)	11,044 (0.18)	621 (0.01)	10,819 (0.18)
**Diabetes without Complications**									
**No**	53,213 (0.30)	1601 (0.01)	68,853 (0.39)	29,968 (0.26)	686 (0.01)	49,799 (0.44)	23,245 (0.38)	915 (0.01)	19,054 (0.31)
**Yes**	23,256 (0.13)	688 (0.00)	26,887 (0.15)	13,121 (0.12)	300 (0.00)	19,410 (0.17)	10,135 (0.17)	388 (0.01)	7477 (0.12)
**Congestive Heart Failure**									
**No**	59,615 (0.34)	1422 (0.01)	67,193 (0.39)	33,447 (0.30)	628 (0.01)	48,697 (0.43)	26,168 (0.43)	794 (0.01)	18,496 (0.30)
**Yes**	16,854 (0.10)	867 (0.00)	28,547 (0.16)	9642 (0.09)	358 (0.00)	20,512 (0.18)	7212 (0.12)	509 (0.01)	8035 (0.13)
**Acute Renal Failure**									
**No**	65,731 (0.38)	1617 (0.01)	77,179 (0.44)	37,012 (0.33)	698 (0.01)	55,983 (0.49)	28,719 (0.47)	919 (0.02)	21,196 (0.35)
**Yes**	10,738 (0.06)	672 (0.00)	18,561 (0.11)	6077 (0.05)	288 (0.00)	13,226 (0.12)	4661 (0.08)	384 (0.01)	5335 (0.09)
**Chronic Obstructive Pulmonary Disease**									
**No**	65,273 (0.37)	1736 (0.01)	78,892 (0.45)	36,725 (0.32)	751 (0.01)	56,980 (0.50)	28,548 (0.47)	985 (0.02)	21,912 (0.36)
**Yes**	11,196 (0.06)	553 (0.00)	16,848 (0.10)	6364 (0.06)	235 (0.00)	12,229 (0.11)	4832 (0.08)	318 (0.01)	4619 (0.08)
**Vascular Disease**									
**No**	67,123 (0.38)	1918 (0.01)	82,866 (0.47)	37,732 (0.33)	823 (0.01)	59,891 (0.53)	29,391 (0.48)	1095 (0.02)	22,975 (0.38)
**Yes**	9346 (0.05)	371 (0.00)	12,874 (0.07)	5357 (0.05)	163 (0.00)	9318 (0.08)	3989 (0.07)	208 (0.00)	3556 (0.06)
**Diabetes with Chronic Complications**									
**No**	69,908 (0.40)	2004 (0.01)	87,549 (0.50)	39,152 (0.35)	858 (0.01)	63,351 (0.56)	30,756 (0.50)	1146 (0.02)	24,198 (0.40)
**Yes**	6561 (0.04)	285 (0.00)	8191 (0.05)	3937 (0.03)	128 (0.00)	5858 (0.05)	2624 (0.04)	157 (0.00)	2333 (0.04)
**Seizure Disorders and Convulsions**									
**No**	71,994 (0.41)	1996 (0.01)	87,469 (0.50)	40,527 (0.36)	848 (0.01)	63,312 (0.56)	31,467 (0.51)	1148 (0.02)	24,157 (0.39)
**Yes**	4475 (0.03)	293 (0.00)	8271 (0.05)	2562 (0.02)	138 (0.00)	5897 (0.05)	1913 (0.03)	155 (0.00)	2374 (0.04)
**Cardio-Respiratory Failure and Shock**									
**No**	72,323 (0.41)	1650 (0.01)	88,099 (0.50)	40,772 (0.36)	711 (0.01)	63,792 (0.56)	31,551 (0.52)	939 (0.02)	24,307 (0.40)
**Yes**	4146 (0.02)	639 (0.00)	7641 (0.04)	2317 (0.02)	275 (0.00)	5417 (0.05)	1829 (0.03)	364 (0.01)	2224 (0.04)

Numbers listed are n (%) by facility type, *IRF* inpatient rehabilitation facility, *LTCH* long-term care hospital, *SNF* skilled nursing facility, *LOS* length of stay, *ICU* intensive care unit, *CCU* coronary care unit

**Fig. 3:**
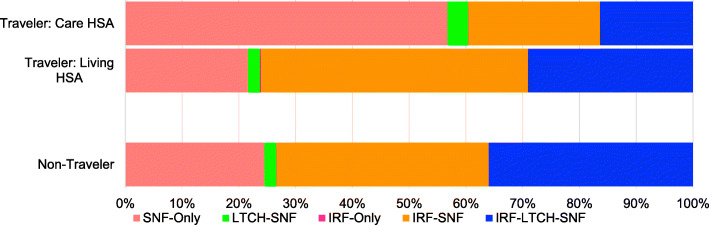
Hospital Service Area combinations by traveling status for stroke rehabilitation. The first and second “traveler” bars represent the facility combinations in the HSAs where the patient received care and where the patient lived, respectively. The “non-traveler” bar represents the facility combination availability for patients who received care in the HSA in which they live. For those who travel, the majority live in IRF-SNF HSAs (second bar, orange section) and travel to SNF-only HSAs to receive care (first bar, pink section). *HSA* Hospital Service Area, *Care HSA* HSA in which patient received care, *Living HSA* HSA in which patient resided, *IRF* inpatient rehabilitation facility, *LTCH* long-term care hospital, *SNF* skilled nursing facility

Approximately 65% of patients (113,248) received care in the HSA where they lived and were termed “non-travelers”. Figure 3 shows the facility combinations for non-travelers and compares them to the facility combinations experienced by “travelers”—patients who live in one HSA and receive care in another (about 35% of all patients). For non-travelers, about 37.4% lived and received care in IRF-SNF HSAs and 35.9% in IRF-LTCH-SNF HSAs. Approximately 79.4% of travelers who lived in a SNF-only HSA traveled to IRF-SNF (49.9%) or IRF-LTCH-SNF HSAs (29.5%) for care (45.1% of all travelers). It is possible for a beneficiary to live in an HSA with one combination of facilities and travel to another HSA with the same combination. For example, in this study 6432 (10.5% of all patients) lived in a SNF-only HSA and traveled to a SNF-only HSA for care.

### Logistic regression

Table 2 presents the results of a multivariable model regressing traveling status (yes/no) on the combination of facility types available where the patient lives, controlling for demographic and clinical covariates. Additional regression results of the remaining HCCs can be found in the supplemental material [see Additional file 2]. These results indicated that patients who lived in any other HSA combination besides IRF-LTCH-SNF had significantly higher odds of traveling compared to the IRF-LTCH-SNF combination (SNF-only OR: 5.44, 95% confidence interval: 5.32, 5.57; LTCH-SNF OR: 4.04, 95% CI: 3.84, 4.26; IRF-only OR: 6.80, 95% CI: 3.75, 12.30; IRF-SNF OR: 1.40, 95% CI: 1.36, 1.43). However, we are cautious about the interpretation of the significant IRF-only finding as only 0.02% of patients live in such an HSA. Relative to individuals 65–69 years old, higher age was significantly associated with lower odds of traveling (OR range: 0.62–0.94); the 21–64 age group was not significantly associated with traveling compared to the 65–69 age group. Additionally, being female (OR: 0.96, 95% CI: 0.94, 0.98) and being dual eligible (OR: 0.77, 95% CI: 0.75, 0.79) were significantly associated with lower odds of traveling, controlling for all other characteristics. Conversely, minority status (Hispanic OR: 1.14, 95% CI: 1.09–1.89; black OR: 1.06, 95% CI: 1.03, 1.09; other OR: 1.33, 95% CI: 1.27, 1.40) and having an ICU/CCU stay (OR: 1.26, 95% CI: 1.24, 1.28) were significantly associated with higher odds of traveling, controlling for all other variables.

**Table 2:** Regression results regressing traveling status on facility type combinations available where the patient lived (*n* = 174,498)

Variables	OR	Lower Bound, 95% CI	Upper Bound, 95% CI	p
*Living Combination				
**IRF-LTCH-SNF**	Ref.			
**SNF-only**	5.443	5.318	5.571	< 0.001
**LTCH-SNF**	4.043	3.835	4.262	< 0.001
**IRF-only**	6.796	3.754	12.303	< 0.001
**IRF-SNF**	1.397	1.363	1.432	< 0.001
*Age, yrs				
**21–64**	0.986	0.942	1.033	0.621
**65–69**	Ref.			
**70–74**	0.936	0.901	0.971	0.003
**75–79**	0.856	0.825	0.887	< 0.001
**80–84**	0.727	0.702	0.754	< 0.001
**85+**	0.622	0.601	0.643	< 0.001
*Race				
**Non-Hispanic White**	Ref.			
**Non-Hispanic Black**	1.058	1.027	1.089	0.001
**Hispanic**	1.138	1.090	1.188	< 0.001
**Other**	1.333	1.269	1.401	< 0.001
*Sex				
**Male**	Ref.			
**Female**	0.960	0.942	0.978	< 0.001
**Stroke Type**				
**Ischemic**	Ref.			
**Hemorrhagic**	1.012	0.983	1.043	0.493
*Acute LOS, days				
**0–3**	Ref.			
**4–7**	1.030	1.010	1.051	0.015
**8–11**	1.032	1.000	1.065	0.098
**12–25**	1.089	1.045	1.135	0.001
**26+**	1.185	1.038	1.353	0.035
*Dual Eligible				
**No**	Ref.			
**Yes**	0.769	0.752	0.787	< 0.001
*ICU/CCU				
**No**	Ref.			
**Yes**	1.259	1.236	1.283	< 0.001
*Hemiplegia/Hemiparesis	1.191	1.169	1.213	< 0.001
**Specified Heart Arrhythmias**	0.998	0.978	1.018	0.856
*Diabetes without Complications	0.969	0.949	0.989	0.011
**Congestive Heart Failure**	0.982	0.960	1.004	0.176
*Acute Renal Failure	1.041	1.015	1.068	0.008
*Chronic Obstructive Pulmonary Disease	0.920	0.897	0.943	< 0.001
**Vascular Disease**	0.994	0.967	1.022	0.715
*Diabetes with Chronic Complications	0.935	0.903	0.969	0.002
*Seizure Disorders and Convulsions	0.916	0.885	0.948	< 0.001
**Cardio-Respiratory Failure and Shock**	1.003	0.966	1.042	0.881

*Significance at α < 0.05, *IRF* inpatient rehabilitation facility, *LTCH* long-term care hospital, *SNF* skilled nursing facility, *LOS* length of stay, *ICU* intensive care unit, *CCU* coronary care unit

## Discussion

This study uniquely identified patterns of rehabilitation services use and described the “traveler effect”—patients who lived in one HSA and traveled to another to receive care—for Medicare patients with stroke. Geographically, the vast majority of HSAs in the US that provided rehabilitation services for stroke were SNF-only. However, about one-third of patients with stroke lived in SNF-only HSAs, and over 35% traveled to receive care. So why were patients traveling? For stroke, almost 99% of patients received care in SNFs and IRFs. The 2019 MedPAC report stated that beneficiaries’ access to IRF services remains adequate [23] and access to SNF services is adequate for most beneficiaries [24]. In fact, the report cites less than 1% of beneficiaries live in a county without a SNF [24]. These conclusions were similar in the MedPAC reports describing 2013 and 2014 data [25, 26]. However, assessments of access to care may not take into consideration if care (e.g., facility type or quality) exists where beneficiaries live—only that the facility exists. We saw examples of this as some beneficiaries traveled from their home HSA to an HSA with the same facility combination (e.g., living in a SNF-only HSA and traveling to a different SNF-only HSA). Therefore, this may be indicative of insufficient care at the HSA level. Although the 2013 report examined acute care services, the National Academies of Science, Engineering, and Medicine indicated that variation in utilization continues to exist at the HSA level [27]. This is likely also true for PAC, to which over 73% of the variation in Medicare spending can be attributed [27].

The findings suggested that living in an area that does not have all three facility types available is associated with higher odds of traveling to receive PAC. This may be due to myriad factors both observable and unobservable, such as the level of care prescribed, market availability, distance to facility, or patient preference. In general, IRF care provides more intensive rehabilitation than SNF, and there are substantially fewer IRFs than SNFs in the US. Although care in this population was predominantly split between those two facility types, less than 20% of the HSAs in the US provided both IRF and SNF care, suggesting that market availability may be an issue. Additionally, a previous study by Buntin et al. showed IRF care to be superior to SNF care for the average Medicare patient for stroke PAC in terms of patient outcomes such as mortality and return to community [16]. In that study, the authors adjusted for both observable (e.g., demographics, severity) and unobservable characteristics such as a patient’s proximity to PAC providers and availability of provider types near the patient [16]. Therefore, in the current study, we believe there may be unobservable factors associated with HSAs which contain all three facility types that are associated with lower odds of traveling.

Results from the regression analysis suggested that patients were more likely to travel if they were a minority race. This finding may be described by many possible factors, including social risk factors highlighted in the 2016 ASPE Report to Congress. First, it is important to consider what the meaning of the race variable may encompass; it is likely not just a group of individuals with similar physical traits. Race as captured in this study may also be indicative of other social risk factors such as income level, housing stability, household size, discrimination, and distribution of resources. Historical and present discrimination and segregation in housing among minority races may dictate where an individual of a minority race lives [28]; that area may not have a sufficient number of facilities or may lack quality care. Therefore, this may result in more frequent travel for those individuals to receive levels of prescribed care. Secondly, individuals of a racial minority may have worse health and may require increased levels of PAC than their non-Hispanic white counterparts. In a different Medicare records study on older individuals with hip fractures, Graham et al. found lower levels of functional status in Hispanics and non-Hispanic blacks compared to non-Hispanic whites [29]. This may suggest that these racial minorities may require more intensive care. As more intensive care is provided in IRFs compared to SNFs, and the nation is predominantly SNF-dense, it is logical to associate higher levels of required care with traveling to an HSA that provides more intensive services. This finding concerning the impact of race on traveling patterns aligns with the first of three strategies outlined in the 2020 ASPE Report to Congress to measure and report social risk factors in order to develop fair quality standards and support beneficiaries with social risk factors [30].

This study had several limitations including the exclusion of home health services, the determination of PAC setting, the method by which facility combinations were determined, and the way traveling status was identified. Concerning the exclusion of home health, the provision of services by one home health agency may not be limited to one HSA; a single home health agent may travel to multiple HSAs to provide care to patients in the patients’ homes. Therefore, the care location of a home health facility is not comparable to the care location of other rehabilitation service types. However, even though patients receive services in the HSA where they live (in their home), many “non-travelers” who utilize home health are excluded. Next, this study only examined if beneficiaries traveled outside their HSA to their first site of rehabilitation, which may not have been reflective of the final, intended destination of care [20, 31]. Future studies should examine whether transitions of care occur more often for those who travel compared to those who receive care in their HSA. Additionally, the method by which we determined the combination of available rehabilitation facility types was patient based. We calculated the number of patients seen in each HSA by facility type (i.e., IRF, LTCH, SNF) from 2013 to 2014. If over these 2 years no patients were seen in a certain facility type, we assumed that facility type did not exist or did not provide stroke rehabilitation. As this study covers 2 years of 100% Medicare records, we believe this assumption to be true in the vast majority of HSAs. Finally, patients were identified as travelers if the HSA number in which they lived did not match the HSA number in which they received care; distance of travel was not considered. It is possible that individuals who lived and received care in the same HSA may have traveled a farther distance for care compared to another patient who lived in one HSA and traveled to another HSA for care. This would depend on the size of the HSA and/or the patient’s geographic location within an HSA region, neither of which were taken into consideration in the current study.

To better inform policy initiatives, we need to further understand why and under what circumstances traveling occurs. Current Bundled Payments for Care Improvement Initiative (BPCI) Models 2–4 do not consider the traveler effect. Distance that patients travel to receive services is not part of the calculation formula in any BPCI Model [32]. Given the goal of BPCI is to link payments for all providers during an episode of care, we suggest CMS examine the traveler effect in BPCI Models to avoid under- or over-estimate of Medicare expenditures and also to ensure quality of continuous care for patients. Similarly, VBP programs examine non-clinical factors and social risk factors that influence service use and health outcomes [14, 15], and our findings suggest that traveling distance is one that warrants further research. The findings from our study can also be used by social workers and discharge planners involved in transitions of care to better understand factors associated with traveling for care.

## Conclusions

This study concludes that from 2013 to 2014, the majority of the nation’s HSAs where stroke patients received rehabilitative care were SNF-only. However, only about one-third of patients with stroke lived in SNF-only HSAs, and over 35% traveled to receive care. This study provides a foundation on which to better quantify drivers of geographic variation in PAC use across the US. More specifically, this study presents previously undescribed drivers variation in PAC service utilization among Medicare beneficiaries—the “traveler effect”. Future research should explore factors that influence “traveling” such as quality or density of available facilities in an area to optimize patients’ outcomes. In regard to political implementation, the availability of facility types where a patient lives may influence the level of care prescribed, the level of care received, and whether a patient travels for care. Current and future healthcare policy such as VBP programs should consider the determinants and effects of the “traveler effect” on Medicare beneficiaries. This may uncover previously undescribed drivers of geographic variation in PAC.

## Supplementary Information

**Additional file 1.** Additional clinical characteristics by post-acute care service type and traveling status. This table shows the remaining clinical characteristics (Hierarchical Condition Categories) present in the data.

**Additional file 2. **Additional logistic regression results regressing traveling status on the combination of facility types available where the patient lived, controlling for demographic and clinical variables (*n* = 174,498); includes remaining Hierarchical Condition Categories.

## Data Availability

The data that support the findings of this study are available from the Centers for Medicare and Medicaid Services (CMS) but restrictions apply to the availability of these data, which were used under a data use agreement for the study, and so are not publicly available. Data are available from CMS through the Research Data Assistance Center (ResDAC). The ZIP code to HSA crosswalks analyzed during the current study are available from Dartmouth Atlas, https://atlasdata.dartmouth.edu/static/supp_research_data#crosswalks.

## Abbreviations

AIDS: Acquired immunodeficiency syndrome
ASPE: Office of the Assistant Secretary for Planning and Evaluation
CCU: Coronary care unit
CI: Confidence interval
DRG: Diagnosis-related group
HCC: Hierarchical Condition Category
HHA: Home health agency
HIV: Human immunodeficiency virus
HSA: Hospital Service Area
ICU: Intensive care unit
IRF: Inpatient rehabilitation facility
LOS: Length of stay
LTCH: Long-term care hospital
MedPAC: Medicare Payment Advisory Commission
OR: Odds ratio
PAC: Post-acute care
SNF: Skilled nursing facility
US: United States
VBP: Value-Based Purchasing
