# Supplementary material for: Post-acute care use patterns among Hospital Service Areas by older adults in the United States: a cross-sectional study
Source: BMC Health Serv Res. 2021 Feb 25;21:176. doi: 10.1186/s12913-021-06159-z (PMC7905663; doi:10.1186/s12913-021-06159-z)
Supplement: Supplementary file 1 — Additional file 1. Additional clinical characteristics by post-acute care service type and traveling status. This table shows the remaining clinical characteristics (Hierarchical Condition Categories) present in the data. [file 12913_2021_6159_MOESM1_ESM.docx]

Additional File 1. Additional clinical characteristics by post-acute care service type and traveling status.

|  | **TOTAL COHORT** | | | **NON-TRAVELERS** | | | **TRAVELERS** | | |
| --- | --- | --- | --- | --- | --- | --- | --- | --- | --- |
|  | **N=174498** | | | **N=113284** | | | **N=61214** | | |
| **Variables** | **IRF (n=76469)** | **LTCH  (n=2289)** | **SNF  (n=95740)** | **IRF  (n=43089)** | **LTCH  (n=986)** | **SNF  (n=69209)** | **IRF  (n=33380)** | **LTCH  (n=1303)** | **SNF  (n=26531)** |
| **HIV/AIDS** |  |  |  |  |  |  |  |  |  |
| **No** | 76302 (0.44) | 2286 (0.01) | 95612 (0.55) | 42987 (0.38) | 984 (0.01) | 69117 (0.61) | 33315 (0.54) | 1302 (0.02) | 26495 (0.43) |
| **Yes** | 167 (0.00) | 3 (0.00) | 128 (0.00) | 102 (0.00) | 2 (0.00) | 92 (0.00) | 65 (0.00) | 1 (0.00) | 36 (0.00) |
| **Septicemia, Sepsis, Systemic  Inflammatory Response Syndrome/Shock** |  |  |  |  |  |  |  |  |  |
| **No** | 74377 (0.43) | 1951 (0.01) | 90420 (0.52) | 41909 (0.37) | 841 (0.01) | 65442 (0.58) | 32468 (0.53) | 1110 (0.02) | 24978 (0.41) |
| **Yes** | 2092 (0.01) | 338 (0.00) | 5320 (0.03) | 1180 (0.01) | 145 (0.00) | 3767 (0.03) | 912 (0.01) | 193 (0.00) | 1553 (0.03) |
| **Opportunistic Infections** |  |  |  |  |  |  |  |  |  |
| **No** | 76320 (0.44) | 2274 (0.01) | 95474 (0.55) | 43010 (0.38) | 979 (0.01) | 69021 (0.61) | 33310 (0.54) | 1295 (0.02) | 26453 (0.43) |
| **Yes** | 149 (0.00) | 15 (0.00) | 266 (0.00) | 79 (0.00) | 7 (0.00) | 188 (0.00) | 70 (0.00) | 8 (0.00) | 78 (0.00) |
| **Metastatic Cancer and Acute  Leukemia** |  |  |  |  |  |  |  |  |  |
| **No** | 75553 (0.43) | 2237 (0.01) | 94225 (0.54) | 42587 (0.38) | 963 (0.01) | 68165 (0.60) | 32966 (0.54) | 1274 (0.02) | 26060 (0.43) |
| **Yes** | 916 (0.01) | 52 (0.00) | 1515 (0.01) | 502 (0.00) | 23 (0.00) | 1044 (0.01) | 414 (0.01) | 29 (0.00) | 471 (0.01) |
| **Lung and Other Severe  Cancers** |  |  |  |  |  |  |  |  |  |
| **No** | 75824 (0.43) | 2262 (0.01) | 94772 (0.54) | 42708 (0.38) | 976 (0.01) | 68504 (0.60) | 33116 (0.54) | 1286 (0.02) | 26268 (0.43) |
| **Yes** | 645 (0.00) | 27 (0.00) | 968 (0.01) | 381 (0.00) | 10 (0.00) | 705 (0.01) | 264 (0.00) | 17 (0.00) | 263 (0.00) |
| **Lymphoma and Other Cancers** |  |  |  |  |  |  |  |  |  |
| **No** | 75726 (0.43) | 2262 (0.01) | 94644 (0.54) | 42677 (0.38) | 972 (0.01) | 68431 (0.60) | 33049 (0.54) | 1290 (0.02) | 26213 (0.43) |
| **Yes** | 743 (0.00) | 27 (0.00) | 1096 (0.01) | 412 (0.00) | 14 (0.00) | 778 (0.01) | 331 (0.01) | 13 (0.00) | 318 (0.01) |
| **Colorectal, Bladder, and  Other Cancers** |  |  |  |  |  |  |  |  |  |
| **No** | 76044 (0.44) | 2269 (0.01) | 95065 (0.54) | 42833 (0.38) | 977 (0.01) | 68719 (0.61) | 33211 (0.54) | 1292 (0.02) | 26346 (0.43) |
| **Yes** | 425 (0.00) | 20 (0.00) | 675 (0.00) | 256 (0.00) | 9 (0.00) | 490 (0.00) | 169 (0.00) | 11 (0.00) | 185 (0.00) |
| **Breast, Prostate, and Other  Cancers and Tumors** |  |  |  |  |  |  |  |  |  |
| **No** | 74778 (0.43) | 2242 (0.01) | 93546 (0.54) | 42108 (0.37) | 970 (0.01) | 67640 (0.60) | 32670 (0.53) | 1272 (0.02) | 25906 (0.42) |
| **Yes** | 1691 (0.01) | 47 (0.00) | 2194 (0.01) | 981 (0.01) | 16 (0.00) | 1569 (0.01) | 710 (0.01) | 31 (0.00) | 625 (0.01) |
| ***Diabetes with Acute  Complications** |  |  |  |  |  |  |  |  |  |
| **No** | 76114 (0.44) | 2262 (0.01) | 95155 (0.55) | 42884 (0.38) | 975 (0.01) | 68783 (0.61) | 33230 (0.54) | 1287 (0.02) | 26372 (0.43) |
| **Yes** | 355 (0.00) | 27 (0.00) | 585 (0.00) | 205 (0.00) | 11 (0.00) | 426 (0.00) | 150 (0.00) | 16 (0.00) | 159 (0.00) |
| **Protein-Calorie  Malnutrition** |  |  |  |  |  |  |  |  |  |
| **No** | 73594 (0.42) | 1796 (0.01) | 87594 (0.50) | 41424 (0.37) | 754 (0.01) | 63418 (0.56) | 32170 (0.53) | 1042 (0.02) | 24176 (0.39) |
| **Yes** | 2875 (0.02) | 493 (0.00) | 8146 (0.05) | 1665 (0.01) | 232 (0.00) | 5791 (0.05) | 1210 (0.02) | 261 (0.00) | 2355 (0.04) |
| **Morbid Obesity** |  |  |  |  |  |  |  |  |  |
| **No** | 72761 (0.42) | 2117 (0.01) | 91606 (0.52) | 40959 (0.36) | 914 (0.01) | 66259 (0.58) | 31802 (0.52) | 1203 (0.02) | 25347 (0.41) |
| **Yes** | 3708 (0.02) | 172 (0.00) | 4134 (0.02) | 2130 (0.02) | 72 (0.00) | 2950 (0.03) | 1578 (0.03) | 100 (0.00) | 1184 (0.02) |
| **Other Significant Endocrine  and Metabolic Disorders** |  |  |  |  |  |  |  |  |  |
| **No** | 73422 (0.42) | 2114 (0.01) | 91348 (0.52) | 41391 (0.37) | 905 (0.01) | 66064 (0.58) | 32031 (0.52) | 1209 (0.02) | 25284 (0.41) |
| **Yes** | 3047 (0.02) | 175 (0.00) | 4392 (0.03) | 1698 (0.01) | 81 (0.00) | 3145 (0.03) | 1349 (0.02) | 94 (0.00) | 1247 (0.02) |
| **End-Stage Liver Disease** |  |  |  |  |  |  |  |  |  |
| **No** | 76299 (0.44) | 2273 (0.01) | 95400 (0.55) | 42992 (0.38) | 977 (0.01) | 68981 (0.61) | 33307 (0.54) | 1296 (0.02) | 26419 (0.43) |
| **Yes** | 170 (0.00) | 16 (0.00) | 340 (0.00) | 97 (0.00) | 9 (0.00) | 228 (0.00) | 73 (0.00) | 7 (0.00) | 112 (0.00) |
| **Cirrhosis of Liver** |  |  |  |  |  |  |  |  |  |
| **No** | 76212 (0.44) | 2270 (0.01) | 95367 (0.55) | 42952 (0.38) | 983 (0.01) | 68938 (0.61) | 33260 (0.54) | 1287 (0.02) | 26429 (0.43) |
| **Yes** | 257 (0.00) | 19 (0.00) | 373 (0.00) | 137 (0.00) | 3 (0.00) | 271 (0.00) | 120 (0.00) | 16 (0.00) | 102 (0.00) |
| **Chronic Hepatitis** |  |  |  |  |  |  |  |  |  |
| **No** | 76270 (0.44) | 2276 (0.01) | 95535 (0.55) | 42973 (0.38) | 981 (0.01) | 69076 (0.61) | 33297 (0.54) | 1295 (0.02) | 26459 (0.43) |
| **Yes** | 199 (0.00) | 13 (0.00) | 205 (0.00) | 116 (0.00) | 5 (0.00) | 133 (0.00) | 83 (0.00) | 8 (0.00) | 72 (0.00) |
| **Intestinal Obstruction/ Perforation** |  |  |  |  |  |  |  |  |  |
| **No** | 75472 (0.43) | 2171 (0.01) | 93647 (0.54) | 42517 (0.38) | 920 (0.01) | 67737 (0.60) | 32955 (0.54) | 1251 (0.02) | 25910 (0.42) |
| **Yes** | 997 (0.01) | 118 (0.00) | 2093 (0.01) | 572 (0.01) | 66 (0.00) | 1472 (0.01) | 425 (0.01) | 52 (0.00) | 621 (0.01) |
| **Chronic Pancreatitis** |  |  |  |  |  |  |  |  |  |
| **No** | 76351 (0.44) | 2282 (0.01) | 95562 (0.55) | 43028 (0.38) | 984 (0.01) | 69072 (0.61) | 33323 (0.54) | 1298 (0.02) | 26490 (0.43) |
| **Yes** | 118 (0.00) | 7 (0.00) | 178 (0.00) | 61 (0.00) | 2 (0.00) | 137 (0.00) | 57 (0.00) | 5 (0.00) | 41 (0.00) |
| **Inflammatory Bowel  Disease** |  |  |  |  |  |  |  |  |  |
| **No** | 76061 (0.44) | 2271 (0.01) | 95261 (0.55) | 42866 (0.38) | 981 (0.01) | 68859 (0.61) | 33195 (0.54) | 1290 (0.02) | 26402 (0.43) |
| **Yes** | 408 (0.00) | 18 (0.00) | 479 (0.00) | 223 (0.00) | 5 (0.00) | 350 (0.00) | 185 (0.00) | 13 (0.00) | 129 (0.00) |
| **Bone/Joint/Muscle  Infections/Necrosis** |  |  |  |  |  |  |  |  |  |
| **No** | 76072 (0.44) | 2240 (0.01) | 94984 (0.54) | 42861 (0.38) | 965 (0.01) | 68659 (0.61) | 33211 (0.54) | 1275 (0.02) | 26325 (0.43) |
| **Yes** | 397 (0.00) | 49 (0.00) | 756 (0.00) | 228 (0.00) | 21 (0.00) | 550 (0.00) | 169 (0.00) | 28 (0.00) | 206 (0.00) |
| ***Rheumatoid Arthritis and  Inflammatory Connective  Tissue Disease** |  |  |  |  |  |  |  |  |  |
| **No** | 73507 (0.42) | 2195 (0.01) | 91726 (0.53) | 41378 (0.37) | 958 (0.01) | 66224 (0.58) | 32129 (0.52) | 1237 (0.02) | 25502 (0.42) |
| **Yes** | 2962 (0.02) | 94 (0.00) | 4014 (0.02) | 1711 (0.02) | 28 (0.00) | 2985 (0.03) | 1251 (0.02) | 66 (0.00) | 1029 (0.02) |
| **Severe Hematological  Disorders** |  |  |  |  |  |  |  |  |  |
| **No** | 76105 (0.44) | 2272 (0.01) | 95121 (0.55) | 42869 (0.38) | 977 (0.01) | 68762 (0.61) | 33236 (0.54) | 1295 (0.02) | 26359 (0.43) |
| **Yes** | 364 (0.00) | 17 (0.00) | 619 (0.00) | 220 (0.00) | 9 (0.00) | 447 (0.00) | 144 (0.00) | 8 (0.00) | 172 (0.00) |
| **Disorders of Immunity** |  |  |  |  |  |  |  |  |  |
| **No** | 75784 (0.43) | 2246 (0.01) | 94622 (0.54) | 42677 (0.38) | 966 (0.01) | 68399 (0.60) | 33107 (0.54) | 1280 (0.02) | 26223 (0.43) |
| **Yes** | 685 (0.00) | 43 (0.00) | 1118 (0.01) | 412 (0.00) | 20 (0.00) | 810 (0.01) | 273 (0.00) | 23 (0.00) | 308 (0.01) |
| **Coagulation Defects and  Other Specified Hematological  Disorders** |  |  |  |  |  |  |  |  |  |
| **No** | 71756 (0.41) | 2018 (0.01) | 88916 (0.51) | 40473 (0.36) | 866 (0.01) | 64386 (0.57) | 31283 (0.51) | 1152 (0.02) | 24530 (0.40) |
| **Yes** | 4713 (0.03) | 271 (0.00) | 6824 (0.04) | 2616 (0.02) | 120 (0.00) | 4823 (0.04) | 2097 (0.03) | 151 (0.00) | 2001 (0.03) |
| **Schizophrenia** |  |  |  |  |  |  |  |  |  |
| **No** | 76066 (0.44) | 2252 (0.01) | 94665 (0.54) | 42851 (0.38) | 975 (0.01) | 68436 (0.60) | 33215 (0.54) | 1277 (0.02) | 26229 (0.43) |
| **Yes** | 403 (0.00) | 37 (0.00) | 1075 (0.01) | 238 (0.00) | 11 (0.00) | 773 (0.01) | 165 (0.00) | 26 (0.00) | 302 (0.00) |
| **Major Depressive, Bipolar,  and Paranoid Disorders** |  |  |  |  |  |  |  |  |  |
| **No** | 75039 (0.43) | 2229 (0.01) | 93151 (0.53) | 42280 (0.37) | 959 (0.01) | 67397 (0.59) | 32759 (0.54) | 1270 (0.02) | 25754 (0.42) |
| **Yes** | 1430 (0.01) | 60 (0.00) | 2589 (0.01) | 809 (0.01) | 27 (0.00) | 1812 (0.02) | 621 (0.01) | 33 (0.00) | 777 (0.01) |
| **Quadriplegia** |  |  |  |  |  |  |  |  |  |
| **No** | 76393 (0.44) | 2272 (0.01) | 95394 (0.55) | 43047 (0.38) | 977 (0.01) | 68951 (0.61) | 33346 (0.54) | 1295 (0.02) | 26443 (0.43) |
| **Yes** | 76 (0.00) | 17 (0.00) | 346 (0.00) | 42 (0.00) | 9 (0.00) | 258 (0.00) | 34 (0.00) | 8 (0.00) | 88 (0.00) |
| **Paraplegia** |  |  |  |  |  |  |  |  |  |
| **No** | 76340 (0.44) | 2281 (0.01) | 95450 (0.55) | 43014 (0.38) | 980 (0.01) | 68994 (0.61) | 33326 (0.54) | 1301 (0.02) | 26456 (0.43) |
| **Yes** | 129 (0.00) | 8 (0.00) | 290 (0.00) | 75 (0.00) | 6 (0.00) | 215 (0.00) | 54 (0.00) | 2 (0.00) | 75 (0.00) |
| **Spinal Cord Disorders/ Injuries** |  |  |  |  |  |  |  |  |  |
| **No** | 76204 (0.44) | 2281 (0.01) | 95413 (0.55) | 42943 (0.38) | 986 (0.01) | 68972 (0.61) | 33261 (0.54) | 1295 (0.02) | 26441 (0.43) |
| **Yes** | 265 (0.00) | 8 (0.00) | 327 (0.00) | 146 (0.00) | 0 (0.00) | 237 (0.00) | 119 (0.00) | 8 (0.00) | 90 (0.00) |
| **Amyotrophic Lateral Sclerosis  and Other Motor Neuron Disease** |  |  |  |  |  |  |  |  |  |
| **No** | 76431 (0.44) | 2287 (0.01) | 95677 (0.55) | 43065 (0.38) | 985 (0.01) | 69163 (0.61) | 33366 (0.55) | 1302 (0.02) | 26514 (0.43) |
| **Yes** | 38 (0.00) | 2 (0.00) | 63 (0.00) | 24 (0.00) | 1 (0.00) | 46 (0.00) | 14 (0.00) | 1 (0.00) | 17 (0.00) |
| **Cerebral Palsy** |  |  |  |  |  |  |  |  |  |
| **No** | 76409 (0.44) | 2287 (0.01) | 95636 (0.55) | 43055 (0.38) | 984 (0.01) | 69141 (0.61) | 33354 (0.54) | 1303 (0.02) | 26495 (0.43) |
| **Yes** | 60 (0.00) | 2 (0.00) | 104 (0.00) | 34 (0.00) | 2 (0.00) | 68 (0.00) | 26 (0.00) | 0 (0.00) | 36 (0.00) |
| **Myasthenia Gravis/Myoneural  Disorders and Guillain  Barre Syndrome/Inflammatory  and Toxic Neuropathy** |  |  |  |  |  |  |  |  |  |
| **No** | 76216 (0.44) | 2279 (0.01) | 95458 (0.55) | 42944 (0.38) | 982 (0.01) | 68997 (0.61) | 33272 (0.54) | 1297 (0.02) | 26461 (0.43) |
| **Yes** | 253 (0.00) | 10 (0.00) | 282 (0.00) | 145 (0.00) | 4 (0.00) | 212 (0.00) | 108 (0.00) | 6 (0.00) | 70 (0.00) |
| **Muscular Dystrophy** |  |  |  |  |  |  |  |  |  |
| **No** | 76445 (0.44) | 2289 (0.01) | 95704 (0.55) | 43079 (0.38) | 986 (0.01) | 69179 (0.61) | 33366 (0.55) | 1303 (0.02) | 26525 (0.43) |
| **Yes** | 24 (0.00) | 0 (0.00) | 36 (0.00) | 10 (0.00) | 0 (0.00) | 30 (0.00) | 14 (0.00) | 0 (0.00) | 6 (0.00) |
| **Multiple Sclerosis** |  |  |  |  |  |  |  |  |  |
| **No** | 76195 (0.44) | 2281 (0.01) | 95409 (0.55) | 42946 (0.38) | 983 (0.01) | 68978 (0.61) | 33249 (0.54) | 1298 (0.02) | 26431 (0.43) |
| **Yes** | 274 (0.00) | 8 (0.00) | 331 (0.00) | 143 (0.00) | 3 (0.00) | 231 (0.00) | 131 (0.00) | 5 (0.00) | 100 (0.00) |
| **Parkinson's and  Huntington's Diseases** |  |  |  |  |  |  |  |  |  |
| **No** | 74910 (0.43) | 2245 (0.01) | 92741 (0.53) | 42181 (0.37) | 964 (0.01) | 67064 (0.59) | 32729 (0.53) | 1281 (0.02) | 25677 (0.42) |
| **Yes** | 1559 (0.01) | 44 (0.00) | 2999 (0.02) | 908 (0.01) | 22 (0.00) | 2145 (0.02) | 651 (0.01) | 22 (0.00) | 854 (0.01) |
| **Coma, Brain Compression/ Anoxic Damage** |  |  |  |  |  |  |  |  |  |
| **No** | 72100 (0.41) | 1966 (0.01) | 90069 (0.52) | 40971 (0.36) | 865 (0.01) | 65308 (0.58) | 31129 (0.51) | 1101 (0.02) | 24761 (0.40) |
| **Yes** | 4369 (0.03) | 323 (0.00) | 5671 (0.03) | 2118 (0.02) | 121 (0.00) | 3901 (0.03) | 2251 (0.04) | 202 (0.00) | 1770 (0.03) |
| **Respiratory Dependence/ Tracheostomy Status** |  |  |  |  |  |  |  |  |  |
| **No** | 76352 (0.44) | 2220 (0.01) | 95501 (0.55) | 43024 (0.38) | 963 (0.01) | 69054 (0.61) | 33328 (0.54) | 1257 (0.02) | 26447 (0.43) |
| **Yes** | 117 (0.00) | 69 (0.00) | 239 (0.00) | 65 (0.00) | 23 (0.00) | 155 (0.00) | 52 (0.00) | 46 (0.00) | 84 (0.00) |
| **Respiratory Arrest** |  |  |  |  |  |  |  |  |  |
| **No** | 76454 (0.44) | 2287 (0.01) | 95701 (0.55) | 43079 (0.38) | 986 (0.01) | 69180 (0.61) | 33375 (0.55) | 1301 (0.02) | 26521 (0.43) |
| **Yes** | 15 (0.00) | 2 (0.00) | 39 (0.00) | 10 (0.00) | 0 (0.00) | 29 (0.00) | 5 (0.00) | 2 (0.00) | 10 (0.00) |
| **Acute Myocardial  Infarction** |  |  |  |  |  |  |  |  |  |
| **No** | 73845 (0.42) | 2103 (0.01) | 90818 (0.52) | 41689 (0.37) | 908 (0.01) | 65730 (0.58) | 32156 (0.53) | 1195 (0.02) | 25088 (0.41) |
| **Yes** | 2624 (0.02) | 186 (0.00) | 4922 (0.03) | 1400 (0.01) | 78 (0.00) | 3479 (0.03) | 1224 (0.02) | 108 (0.00) | 1443 (0.02) |
| **Unstable Angina and Other  Acute Ischemic Heart Disease** |  |  |  |  |  |  |  |  |  |
| **No** | 75426 (0.43) | 2238 (0.01) | 94127 (0.54) | 42507 (0.38) | 967 (0.01) | 68086 (0.60) | 32919 (0.54) | 1271 (0.02) | 26041 (0.43) |
| **Yes** | 1043 (0.01) | 51 (0.00) | 1613 (0.01) | 582 (0.01) | 19 (0.00) | 1123 (0.01) | 461 (0.01) | 32 (0.00) | 490 (0.01) |
| **Angina Pectoris** |  |  |  |  |  |  |  |  |  |
| **No** | 75984 (0.44) | 2270 (0.01) | 95129 (0.55) | 42809 (0.38) | 979 (0.01) | 68786 (0.61) | 33175 (0.54) | 1291 (0.02) | 26343 (0.43) |
| **Yes** | 485 (0.00) | 19 (0.00) | 611 (0.00) | 280 (0.00) | 7 (0.00) | 423 (0.00) | 205 (0.00) | 12 (0.00) | 188 (0.00) |
| **Cerebral Hemorrhage** |  |  |  |  |  |  |  |  |  |
| **No** | 73154 (0.42) | 2070 (0.01) | 91315 (0.52) | 41399 (0.37) | 910 (0.01) | 66123 (0.58) | 31755 (0.52) | 1160 (0.02) | 25192 (0.41) |
| **Yes** | 3315 (0.02) | 219 (0.00) | 4425 (0.03) | 1690 (0.01) | 76 (0.00) | 3086 (0.03) | 1625 (0.03) | 143 (0.00) | 1339 (0.02) |
| **Ischemic or Unspecified  Stroke** |  |  |  |  |  |  |  |  |  |
| **No** | 72823 (0.42) | 2149 (0.01) | 90792 (0.52) | 41138 (0.36) | 927 (0.01) | 65710 (0.58) | 31685 (0.52) | 1222 (0.02) | 25082 (0.41) |
| **Yes** | 3646 (0.02) | 140 (0.00) | 4948 (0.03) | 1951 (0.02) | 59 (0.00) | 3499 (0.03) | 1695 (0.03) | 81 (0.00) | 1449 (0.02) |
| **Monoplegia, Other Paralytic  Syndromes** |  |  |  |  |  |  |  |  |  |
| **No** | 75749 (0.43) | 2262 (0.01) | 94873 (0.54) | 42703 (0.38) | 977 (0.01) | 68562 (0.61) | 33046 (0.54) | 1285 (0.02) | 26311 (0.43) |
| **Yes** | 720 (0.00) | 27 (0.00) | 867 (0.00) | 386 (0.00) | 9 (0.00) | 647 (0.01) | 334 (0.01) | 18 (0.00) | 220 (0.00) |
| **Atherosclerosis of the  Extremities with Ulceration  or Gangrene** |  |  |  |  |  |  |  |  |  |
| **No** | 76178 (0.44) | 2254 (0.01) | 95042 (0.54) | 42918 (0.38) | 965 (0.01) | 68704 (0.61) | 33260 (0.54) | 1289 (0.02) | 26338 (0.43) |
| **Yes** | 291 (0.00) | 35 (0.00) | 698 (0.00) | 171 (0.00) | 21 (0.00) | 505 (0.00) | 120 (0.00) | 14 (0.00) | 193 (0.00) |
| **Vascular Disease with  Complications** |  |  |  |  |  |  |  |  |  |
| **No** | 75328 (0.43) | 2216 (0.01) | 93933 (0.54) | 42487 (0.38) | 961 (0.01) | 67951 (0.60) | 32841 (0.54) | 1255 (0.02) | 25982 (0.42) |
| **Yes** | 1141 (0.01) | 73 (0.00) | 1807 (0.01) | 602 (0.01) | 25 (0.00) | 1258 (0.01) | 539 (0.01) | 48 (0.00) | 549 (0.01) |
| **Cystic Fibrosis** |  |  |  |  |  |  |  |  |  |
| **No** | 76467 (0.44) | 2289 (0.01) | 95736 (0.55) | 43088 (0.38) | 986 (0.01) | 69207 (0.61) | 33379 (0.55) | 1303 (0.02) | 26529 (0.43) |
| **Yes** | 2 (0.00) | 0 (0.00) | 4 (0.00) | 1 (0.00) | 0 (0.00) | 2 (0.00) | 1 (0.00) | 0 (0.00) | 2 (0.00) |
| **Fibrosis of Lung and  Other Chronic Lung Disorders** |  |  |  |  |  |  |  |  |  |
| **No** | 75826 (0.43) | 2271 (0.01) | 94804 (0.54) | 42706 (0.38) | 979 (0.01) | 68508 (0.60) | 33120 (0.54) | 1292 (0.02) | 26296 (0.43) |
| **Yes** | 643 (0.00) | 18 (0.00) | 936 (0.01) | 383 (0.00) | 7 (0.00) | 701 (0.01) | 260 (0.00) | 11 (0.00) | 235 (0.00) |
| **Aspiration and Specified  Bacterial Pneumonias** |  |  |  |  |  |  |  |  |  |
| **No** | 73878 (0.42) | 1734 (0.01) | 89724 (0.51) | 41705 (0.37) | 750 (0.01) | 64964 (0.57) | 32173 (0.53) | 984 (0.02) | 24760 (0.40) |
| **Yes** | 2591 (0.01) | 555 (0.00) | 6016 (0.03) | 1384 (0.01) | 236 (0.00) | 4245 (0.04) | 1207 (0.02) | 319 (0.01) | 1771 (0.03) |
| **Pneumococcal Pneumonia,  Empyema, Lung Abscess** |  |  |  |  |  |  |  |  |  |
| **No** | 76377 (0.44) | 2277 (0.01) | 95562 (0.55) | 43038 (0.38) | 982 (0.01) | 69074 (0.61) | 33339 (0.54) | 1295 (0.02) | 26488 (0.43) |
| **Yes** | 92 (0.00) | 12 (0.00) | 178 (0.00) | 51 (0.00) | 4 (0.00) | 135 (0.00) | 41 (0.00) | 8 (0.00) | 43 (0.00) |
| **Proliferative Diabetic  Retinopathy and Vitreous  Hemorrhage** |  |  |  |  |  |  |  |  |  |
| **No** | 76411 (0.44) | 2289 (0.01) | 95687 (0.55) | 43051 (0.38) | 986 (0.01) | 69173 (0.61) | 33360 (0.54) | 1303 (0.02) | 26514 (0.43) |
| **Yes** | 58 (0.00) | 0 (0.00) | 53 (0.00) | 38 (0.00) | 0 (0.00) | 36 (0.00) | 20 (0.00) | 0 (0.00) | 17 (0.00) |
| **Exudative Macular Degeneration** |  |  |  |  |  |  |  |  |  |
| **No** | 76453 (0.44) | 2289 (0.01) | 95700 (0.55) | 43079 (0.38) | 986 (0.01) | 69179 (0.61) | 33374 (0.55) | 1303 (0.02) | 26521 (0.43) |
| **Yes** | 16 (0.00) | 0 (0.00) | 40 (0.00) | 10 (0.00) | 0 (0.00) | 30 (0.00) | 6 (0.00) | 0 (0.00) | 10 (0.00) |
| **Dialysis Status** |  |  |  |  |  |  |  |  |  |
| **No** | 75200 (0.43) | 2143 (0.01) | 93931 (0.54) | 42292 (0.37) | 918 (0.01) | 67885 (0.60) | 32908 (0.54) | 1225 (0.02) | 26046 (0.43) |
| **Yes** | 1269 (0.01) | 146 (0.00) | 1809 (0.01) | 797 (0.01) | 68 (0.00) | 1324 (0.01) | 472 (0.01) | 78 (0.00) | 485 (0.01) |
| **Chronic Kidney Disease,  Severe (Stage 5)** |  |  |  |  |  |  |  |  |  |
| **No** | 76177 (0.44) | 2266 (0.01) | 95394 (0.55) | 42907 (0.38) | 976 (0.01) | 68963 (0.61) | 33270 (0.54) | 1290 (0.02) | 26431 (0.43) |
| **Yes** | 292 (0.00) | 23 (0.00) | 346 (0.00) | 182 (0.00) | 10 (0.00) | 246 (0.00) | 110 (0.00) | 13 (0.00) | 100 (0.00) |
| **Chronic Kidney Disease,  Severe (Stage 4)** |  |  |  |  |  |  |  |  |  |
| **No** | 75784 (0.43) | 2277 (0.01) | 94722 (0.54) | 42699 (0.38) | 982 (0.01) | 68440 (0.60) | 33085 (0.54) | 1295 (0.02) | 26282 (0.43) |
| **Yes** | 685 (0.00) | 12 (0.00) | 1018 (0.01) | 390 (0.00) | 4 (0.00) | 769 (0.01) | 295 (0.00) | 8 (0.00) | 249 (0.00) |
| **Pressure Ulcer of Skin with  Necrosis Through to Muscle,  Tendon, or Bone** |  |  |  |  |  |  |  |  |  |
| **No** | 76448 (0.44) | 2269 (0.01) | 95563 (0.55) | 43077 (0.38) | 974 (0.01) | 69083 (0.61) | 33371 (0.55) | 1295 (0.02) | 26480 (0.43) |
| **Yes** | 21 (0.00) | 20 (0.00) | 177 (0.00) | 12 (0.00) | 12 (0.00) | 126 (0.00) | 9 (0.00) | 8 (0.00) | 51 (0.00) |
| **Pressure Ulcer of Skin with  Full Thickness Skin Loss** |  |  |  |  |  |  |  |  |  |
| **No** | 76333 (0.44) | 2229 (0.01) | 94940 (0.54) | 43013 (0.38) | 960 (0.01) | 68645 (0.61) | 33320 (0.54) | 1269 (0.02) | 26295 (0.43) |
| **Yes** | 136 (0.00) | 60 (0.00) | 800 (0.00) | 76 (0.00) | 26 (0.00) | 564 (0.00) | 60 (0.00) | 34 (0.00) | 236 (0.00) |
| **Chronic Ulcer of Skin,  Except Pressure** |  |  |  |  |  |  |  |  |  |
| **No** | 75752 (0.43) | 2234 (0.01) | 94177 (0.54) | 42678 (0.38) | 961 (0.01) | 68092 (0.60) | 33074 (0.54) | 1273 (0.02) | 26085 (0.43) |
| **Yes** | 717 (0.00) | 55 (0.00) | 1563 (0.01) | 411 (0.00) | 25 (0.00) | 1117 (0.01) | 306 (0.00) | 30 (0.00) | 446 (0.01) |
| **Severe Skin Burn or Condition** |  |  |  |  |  |  |  |  |  |
| **No** | 76455 (0.44) | 2288 (0.01) | 95732 (0.55) | 43083 (0.38) | 986 (0.01) | 69203 (0.61) | 33372 (0.55) | 1302 (0.02) | 26529 (0.43) |
| **Yes** | 14 (0.00) | 1 (0.00) | 8 (0.00) | 6 (0.00) | 0 (0.00) | 6 (0.00) | 8 (0.00) | 1 (0.00) | 2 (0.00) |
| **Severe Head Injury** |  |  |  |  |  |  |  |  |  |
| **No** | 76466 (0.44) | 2289 (0.01) | 95730 (0.55) | 43087 (0.38) | 986 (0.01) | 69205 (0.61) | 33379 (0.55) | 1303 (0.02) | 26525 (0.43) |
| **Yes** | 3 (0.00) | 0 (0.00) | 10 (0.00) | 2 (0.00) | 0 (0.00) | 4 (0.00) | 1 (0.00) | 0 (0.00) | 6 (0.00) |
| **Major Head Injury** |  |  |  |  |  |  |  |  |  |
| **No** | 75810 (0.43) | 2249 (0.01) | 94125 (0.54) | 42718 (0.38) | 971 (0.01) | 68103 (0.60) | 33092 (0.54) | 1278 (0.02) | 26022 (0.43) |
| **Yes** | 659 (0.00) | 40 (0.00) | 1615 (0.01) | 371 (0.00) | 15 (0.00) | 1106 (0.01) | 288 (0.00) | 25 (0.00) | 509 (0.01) |
| **Vertebral Fractures without  Spinal Cord Injury** |  |  |  |  |  |  |  |  |  |
| **No** | 75730 (0.43) | 2252 (0.01) | 93907 (0.54) | 42672 (0.38) | 972 (0.01) | 67959 (0.60) | 33058 (0.54) | 1280 (0.02) | 25948 (0.42) |
| **Yes** | 739 (0.00) | 37 (0.00) | 1833 (0.01) | 417 (0.00) | 14 (0.00) | 1250 (0.01) | 322 (0.01) | 23 (0.00) | 583 (0.01) |
| **Hip Fracture/Dislocation** |  |  |  |  |  |  |  |  |  |
| **No** | 75667 (0.43) | 2234 (0.01) | 93146 (0.53) | 42625 (0.38) | 961 (0.01) | 67349 (0.59) | 33042 (0.54) | 1273 (0.02) | 25797 (0.42) |
| **Yes** | 802 (0.00) | 55 (0.00) | 2594 (0.01) | 464 (0.00) | 25 (0.00) | 1860 (0.02) | 338 (0.01) | 30 (0.00) | 734 (0.01) |
| **Traumatic Amputations  and Complications** |  |  |  |  |  |  |  |  |  |
| **No** | 76439 (0.44) | 2287 (0.01) | 95667 (0.55) | 43076 (0.38) | 985 (0.01) | 69163 (0.61) | 33363 (0.55) | 1302 (0.02) | 26504 (0.43) |
| **Yes** | 30 (0.00) | 2 (0.00) | 73 (0.00) | 13 (0.00) | 1 (0.00) | 46 (0.00) | 17 (0.00) | 1 (0.00) | 27 (0.00) |
| **Complications of Specified  Implanted Device or Graft** |  |  |  |  |  |  |  |  |  |
| **No** | 75609 (0.43) | 2203 (0.01) | 93950 (0.54) | 42589 (0.38) | 942 (0.01) | 67926 (0.60) | 33020 (0.54) | 1261 (0.02) | 26024 (0.43) |
| **Yes** | 860 (0.00) | 86 (0.00) | 1790 (0.01) | 500 (0.00) | 44 (0.00) | 1283 (0.01) | 360 (0.01) | 42 (0.00) | 507 (0.01) |
| **Major Organ Transplant  or Replacement Status** |  |  |  |  |  |  |  |  |  |
| **No** | 76268 (0.44) | 2277 (0.01) | 95630 (0.55) | 42996 (0.38) | 982 (0.01) | 69141 (0.61) | 33272 (0.54) | 1295 (0.02) | 26489 (0.43) |
| **Yes** | 201 (0.00) | 12 (0.00) | 110 (0.00) | 93 (0.00) | 4 (0.00) | 68 (0.00) | 108 (0.00) | 8 (0.00) | 42 (0.00) |
| **Artificial Openings for  Feeding or Elimination** |  |  |  |  |  |  |  |  |  |
| **No** | 75861 (0.43) | 2189 (0.01) | 94216 (0.54) | 42723 (0.38) | 948 (0.01) | 68145 (0.60) | 33138 (0.54) | 1241 (0.02) | 26071 (0.43) |
| **Yes** | 608 (0.00) | 100 (0.00) | 1524 (0.01) | 366 (0.00) | 38 (0.00) | 1064 (0.01) | 242 (0.00) | 62 (0.00) | 460 (0.01) |
| **Amputation Status, Lower Limb/ Amputation Complications** |  |  |  |  |  |  |  |  |  |
| **No** | 75660 (0.43) | 2246 (0.01) | 94543 (0.54) | 42607 (0.38) | 963 (0.01) | 68309 (0.60) | 33053 (0.54) | 1283 (0.02) | 26234 (0.43) |
| **Yes** | 809 (0.00) | 43 (0.00) | 1197 (0.01) | 482 (0.00) | 23 (0.00) | 900 (0.01) | 327 (0.01) | 20 (0.00) | 297 (0.00) |

*IRF*, inpatient rehabilitation facility, *LTCH* long-term care hospital, *SNF* skilled nursing facility, *LOS* length of stay, *ICU* intensive care unit,

*CCU* coronary care
