# Supplementary material for: Post-acute care use patterns among Hospital Service Areas by older adults in the United States: a cross-sectional study
Source: BMC Health Serv Res. 2021 Feb 25;21:176. doi: 10.1186/s12913-021-06159-z (PMC7905663; doi:10.1186/s12913-021-06159-z)
Supplement: Supplementary file 2 — Additional file 2. Additional logistic regression results regressing traveling status on the combination of facility types available where the patient lived, controlling for demographic and clinical variables (n = 174,498); includes remaining Hierarchical Condition Categories. [file 12913_2021_6159_MOESM2_ESM.docx]

Additional File 2. Additional logistic regression results regressing traveling status on the combination of facility types available where the patient lives, controlling for demographic and clinical variables (n=174,498); includes remaining hierarchical condition categories.

| **Variables** | **OR** | **95%** | **CI** | **p** |
| --- | --- | --- | --- | --- |
| **HIV/AIDS** | 0.998 | 0.805 | 1.237 | 0.988 |
| **Septicemia, Sepsis,  Systemic Inflammatory Response Syndrome/Shock** | 0.969 | 0.926 | 1.015 | 0.262 |
| **Opportunistic Infections** | 1.100 | 0.920 | 1.315 | 0.382 |
| **Metastatic Cancer and  Acute Leukemia** | 1.026 | 0.952 | 1.106 | 0.568 |
| **Lung and Other Severe Cancers** | 0.910 | 0.829 | 0.999 | 0.096 |
| **Lymphoma and Other Cancers** | 1.003 | 0.921 | 1.094 | 0.948 |
| **Colorectal, Bladder, and  Other Cancers** | 0.892 | 0.796 | 0.998 | 0.095 |
| **Breast, Prostate, and Other  Cancers and Tumors** | 1.005 | 0.947 | 1.067 | 0.883 |
| ***Diabetes with Acute Complications** | 0.831 | 0.735 | 0.938 | 0.012 |
| **Protein-Calorie Malnutrition** | 0.959 | 0.923 | 0.995 | 0.065 |
| **Morbid Obesity** | 0.973 | 0.931 | 1.017 | 0.308 |
| **Other Significant Endocrine  and Metabolic Disorders** | 0.995 | 0.951 | 1.041 | 0.853 |
| **End-Stage Liver Disease** | 1.083 | 0.921 | 1.273 | 0.417 |
| **Cirrhosis of Liver** | 1.047 | 0.906 | 1.209 | 0.604 |
| **Chronic Hepatitis** | 1.178 | 0.986 | 1.408 | 0.131 |
| **Intestinal Obstruction/Perforation** | 0.976 | 0.913 | 1.044 | 0.560 |
| **Chronic Pancreatitis** | 0.975 | 0.787 | 1.207 | 0.844 |
| **Inflammatory Bowel Disease** | 1.032 | 0.913 | 1.167 | 0.673 |
| **Bone/Joint/Muscle Infections/ Necrosis** | 1.010 | 0.900 | 1.133 | 0.890 |
| ***Rheumatoid Arthritis and  Inflammatory Connective Tissue Disease** | 0.910 | 0.869 | 0.952 | 0.001 |
| **Severe Hematological Disorders** | 0.933 | 0.827 | 1.052 | 0.339 |
| **Disorders of Immunity** | 0.918 | 0.839 | 1.006 | 0.122 |
| ***Coagulation Defects and Other  Specified Hematological Disorders** | 1.051 | 1.014 | 1.089 | 0.023 |
| **Schizophrenia** | 0.933 | 0.846 | 1.029 | 0.243 |
| **Major Depressive, Bipolar, and  Paranoid Disorders** | 1.022 | 0.963 | 1.084 | 0.546 |
| **Quadriplegia** | 0.860 | 0.715 | 1.034 | 0.178 |
| **Paraplegia** | 0.870 | 0.723 | 1.048 | 0.218 |
| **Spinal Cord Disorders/Injuries** | 0.992 | 0.854 | 1.152 | 0.928 |
| **Amyotrophic Lateral Sclerosis  and Other Motor Neuron Disease** | 0.795 | 0.546 | 1.156 | 0.313 |
| **Cerebral Palsy** | 1.132 | 0.853 | 1.501 | 0.471 |
| **Myasthenia Gravis/Myoneural Disorders  and Guillain Barre Syndrome/Inflammatory  and Toxic Neuropathy** | 0.979 | 0.835 | 1.149 | 0.830 |
| **Muscular Dystrophy** | 0.705 | 0.434 | 1.142 | 0.233 |
| **Multiple Sclerosis** | 1.008 | 0.870 | 1.167 | 0.933 |
| **Parkinson's and Huntington's Diseases** | 0.943 | 0.892 | 0.998 | 0.086 |
| ***Coma, Brain Compression/Anoxic Damage** | 1.135 | 1.091 | 1.181 | <0.001 |
| ***Respiratory Dependence/Tracheostomy  Status** | 1.302 | 1.091 | 1.553 | 0.014 |
| **Respiratory Arrest** | 0.839 | 0.503 | 1.398 | 0.571 |
| **Acute Myocardial Infarction** | 1.022 | 0.978 | 1.068 | 0.413 |
| **Unstable Angina and Other Acute  Ischemic Heart Disease** | 1.069 | 0.995 | 1.148 | 0.124 |
| **Angina Pectoris** | 1.084 | 0.971 | 1.210 | 0.230 |
| ***Cerebral Hemorrhage** | 1.093 | 1.048 | 1.141 | 0.001 |
| **Ischemic or Unspecified Stroke** | 1.023 | 0.983 | 1.066 | 0.351 |
| **Monoplegia, Other Paralytic Syndromes** | 1.023 | 0.932 | 1.122 | 0.689 |
| **Atherosclerosis of the Extremities  with Ulceration or Gangrene** | 0.885 | 0.781 | 1.002 | 0.106 |
| ***Vascular Disease with Complications** | 1.084 | 1.013 | 1.160 | 0.050 |
| **Cystic Fibrosis** | 1.441 | 0.335 | 6.197 | 0.680 |
| **Fibrosis of Lung and Other Chronic  Lung Disorders** | 0.897 | 0.815 | 0.986 | 0.060 |
| **Aspiration and Specified Bacterial  Pneumonias** | 0.960 | 0.920 | 1.001 | 0.110 |
| **Pneumococcal Pneumonia, Empyema,  Lung Abscess** | 0.820 | 0.655 | 1.028 | 0.149 |
| **Proliferative Diabetic Retinopathy  and Vitreous Hemorrhage** | 1.081 | 0.758 | 1.541 | 0.719 |
| **Exudative Macular Degeneration** | 0.867 | 0.518 | 1.451 | 0.649 |
| **Dialysis Status** | 0.932 | 0.866 | 1.002 | 0.109 |
| **Chronic Kidney Disease, Severe  (Stage 5)** | 0.884 | 0.764 | 1.023 | 0.166 |
| **Chronic Kidney Disease, Severe  (Stage4)** | 0.923 | 0.843 | 1.012 | 0.152 |
| **Pressure Ulcer of Skin with Necrosis  Through to Muscle, Tendon, or Bone** | 0.956 | 0.737 | 1.239 | 0.774 |
| **Pressure Ulcer of Skin with Full  Thickness Skin Loss** | 0.979 | 0.868 | 1.104 | 0.774 |
| **Chronic Ulcer of Skin, Except Pressure** | 0.991 | 0.915 | 1.073 | 0.845 |
| **Severe Skin Burn or Condition** | 1.595 | 0.773 | 3.289 | 0.289 |
| **Severe Head Injury** | 2.270 | 0.846 | 6.096 | 0.172 |
| **Major Head Injury** | 1.042 | 0.963 | 1.126 | 0.390 |
| ***Vertebral Fractures without  Spinal Cord Injury** | 1.144 | 1.063 | 1.231 | 0.002 |
| **Hip Fracture/Dislocation** | 0.936 | 0.877 | 0.999 | 0.096 |
| ***Traumatic Amputations and  Complications** | 1.544 | 1.090 | 2.188 | 0.040 |
| **Complications of Specified  Implanted Device or Graft** | 0.932 | 0.866 | 1.004 | 0.117 |
| ***Major Organ Transplant or  Replacement Status** | 1.631 | 1.337 | 1.990 | <0.001 |
| **Artificial Openings for Feeding  or Elimination** | 0.967 | 0.892 | 1.049 | 0.501 |
| ***Amputation Status, Lower Limb/ Amputation Complications** | 0.834 | 0.765 | 0.910 | 0.001 |
| *Significance at α < 0.05 | | | | |
